# Genome sequence of an *Acinetobacter pittii* strain obtained from a red*-*lored parrot with pneumonia

**DOI:** 10.1128/mra.01038-23

**Published:** 2023-12-19

**Authors:** E. Bello-López, A. S. Escobedo-Muñoz, R. Hernández-Castro, M. A. Cevallos

**Affiliations:** 1 Programa de Genómica Evolutiva, Centro de Ciencias Genómicas, Universidad Nacional Autónoma de México, Cuernavaca, Morelos, Mexico; 2 Departmento de Ecología de Patógenos, Hospital General Dr. Manuel Gea González, Ciudad de México, Mexico; The University of Arizona, Tucson, Arizona, USA

**Keywords:** red-lored parrot, *pneumonia*, beta-lactamase, cephalosporin, *bla*ADC

## Abstract

*Acinetobacter pittii* 978-A_19 was obtained from a parrot with pneumonia. It is resistant to ampicillin, carbenicillin, cephalosporins, clindamycin, and trimethoprim + sulfamethoxazole. The genome encodes a new *bla*ADC allele, a *bla*OXA-502 gene, possesses several virulence genes related to adherence and biofilm formation, and has types I, II, and IV secretion systems.

## ANNOUNCEMENT

**Fig 1:**
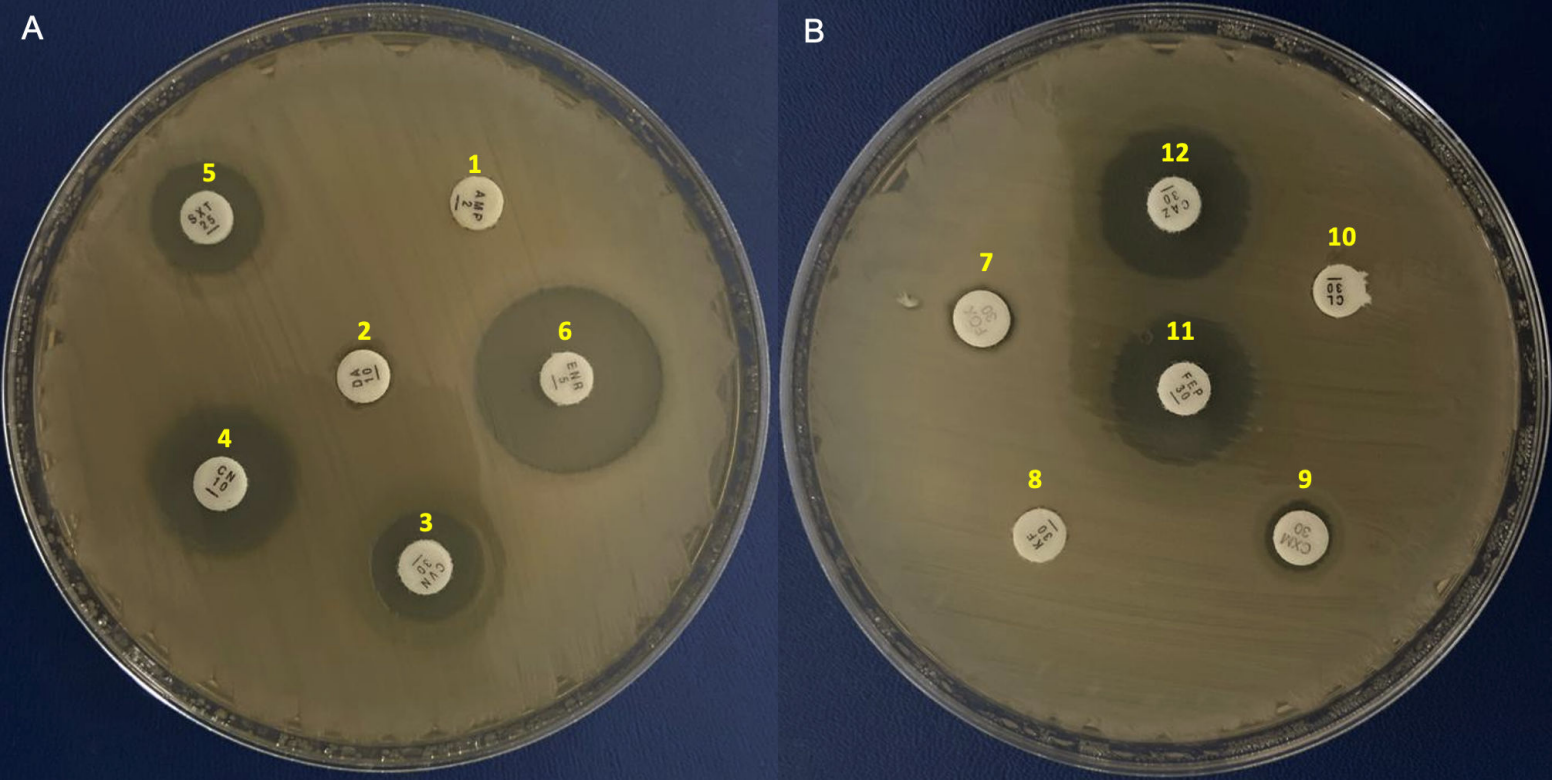
The antimicrobial susceptibility test (antibiogram) of *Acinetobacter pittii* strain on Mueller-Hinton agar. (**A**) Inhibition zone diameters of resistance profile for ampicillin (1, AMP), clindamycin (2, DA), cefovencin (3, CVN), gentamicin (4, CN), and sulfamethoxazole/trimethoprim (5, SXT), and susceptible profile for enrofloxacin (6, ENR). (**B**) Resistance profile for cefoxitin (7, FOX), cephalothin (8, KF), cefuroxime (9, CXM), cephalexin (10, CL), cefepime (11, FEP), and ceftazidime (12, CAZ). 978-A_19 showed resistance to AMP, FEP, CVN, FOX, CXM, CL, KF, CAZ, DA, CN, and SXT, and susceptible profile for ENR.

Strain 978-A_19 was isolated from a red-lored parrot (*Amazona autumnalisis*) with suspected pneumonia and kept in captivity in Mexico City. The veterinarian in charge took the sample introducing sterile cotton swabs into each nasal cavity, rubbed smoothly against the mucosa, and withdrew without touching the skin of the nasal borders. The sample was cultured in MacConkey agar media without antibiotics. The plates were incubated for 24–48 h at 37°C. The strain was purified three consecutive times on MacConkey agar plates from single colonies. To determine the antimicrobial susceptibility, the disk diffusion method was used according to the Clinical and Laboratory Standards Institute (http://em100.edaptivedocs.net/Login.aspx) (Fig. 1).

978-A_19 gDNA was extracted from an overnight culture grown at 37°C and 250 rpm in 5 mL of Luria-Bertani medium (LB) liquid medium, with the Genomic DNA purification kit (Thermo Fisher). Two different platforms were used to obtain its genome sequence. First, a short-read library was constructed using the MGIEasy FS PCR-free DNA library prep set and sequenced (2 × 150 bp) using the BGISEQ-2000 platform. Adapter removal and quality trimming of the DNBseq reads were performed using Trim Galore v0.6.4. Also, a long-read library was constructed following the “Genomic DNA by ligation” protocol (SQK-LSK109; Oxford Nanopore Technologies), and the reads were obtained using a PromethION device. Base calling was performed with Guppy 5.1.13v using the high-accuracy base-calling mode. Adapter sequences were removed using Porechop 0.2.4. Both libraries were constructed and sequenced at the Beijing Genomic Institute (China). To obtain the best genome assembly, short-read assemblies were constructed with three programs: ABySS 2.0.1 (1), SPAdes 3.9.0 (2), and Velvet 1.2.10 (3), with various kmers. Second, long-read assemblies were obtained with Flye 2.7.1-b1590 (4) and Canu 2.1.1 (5). Also, a hybrid assembly was made with Unicycler 0.4.8 (6). Default parameters were used for all software unless otherwise specified. From each program, we selected the assembly with the highest *N*_50_ and the lowest number of contigs to obtain an optimized and merged assembly using Metassembler 1.5 (7). The genome was annotated using the NCBI Prokaryotic Genome Annotation Pipeline (8).(Table 1)

**TABLE 1:** Summary characteristics of whole-genome sequencing of *Acinetobacter pittii* 978-A_19

No. of reads (Illumina)	8,147,394	CDS (with protein)^*a*^	3,750
No. of reads (Oxford Nanopore)/N50	546,426/2,948	rRNAs (5S, 16S, 23S)	6, 6, 6
Assembly *N*_50_ (bp)	2,224,299	tRNAs	73
Contigs/circularized contigs	8/0	ncRNA	4
Largest contig length (bp)	2,224,299	OXA	*bla*OXA-502
Shorter contig length (bp)	35,106	*bla*ADC-292	MDE4038918
Total length (bp)	4,094,008	BioProject	PRJNA937725
Coverage (Oxford Nanopore) Coverage (DNBseq)	362× 297×	BioSample	SAMN33416577
GC content (%)	38.83	SRA (Oxford nanopore) SRA (DNBseq)	SRX22631229 SRX22256086
Genes	3,894	GenBank	JARCHN000000000

^*a*^ CDS: CoDing Sequence.

We identified the antibiotic-resistance genes encoded by this genome with CARD 3.1.4 (9). 978-A_19 possesses a new allele of a *bla*ADC beta-lactamase involved in cephalosporine resistance and a *bla*OXA-502 allele that usually hydrolyzes carbapenems weakly. Additionally, the strain has four efflux bombs AbeS, AdeF, AmvA, and AbaF that, when overexpressed, could lead to a reduction of susceptibility to antibiotics like macrolides, phosphonic acid, fluoroquinolones, and tetracycline.

To identify the genes potentially involved in virulence, we consulted the Virulence Factor Database (10). The strain possesses genes involved in adherence (*ompA*, *filP*, and *pilE*); genes related to biofilm formation like *bap*, polysaccharide poly-N-acetylglucosamine synthesis, and all elements required for *Csu* pili formation. Genome annotations showed that 978-A_19 has the whole gene set for the synthesis of acinetobactin, a molecule required for iron uptake, and the genes encoding for types I, II, and VI secretion systems. These systems have been linked to virulence, but the last is also linked to antibacterial activity (11).

## References

[1] Jackman SD , Vandervalk BP , Mohamadi H , Chu J , Yeo S , Hammond SA , Jahesh G , Khan H , Coombe L , Warren RL , Birol I . 2017. ABySS 2.0: resource-efficient assembly of large genomes using a bloom filter. Genome Res 27:768–777. doi:10.1101/gr.214346.116 28232478 PMC5411771

[2] Bankevich A , Nurk S , Antipov D , Gurevich AA , Dvorkin M , Kulikov AS , Lesin VM , Nikolenko SI , Pham S , Prjibelski AD , Pyshkin AV , Sirotkin AV , Vyahhi N , Tesler G , Alekseyev MA , Pevzner PA . 2012. SPAdes: a new genome assembly algorithm and its applications to single-cell sequencing. J Comput Biol 19:455–477. doi:10.1089/cmb.2012.0021 22506599 PMC3342519

[3] Zerbino DR , Birney E . 2008. Velvet: algorithms for de novo short read assembly using de Bruijn graphs. Genome Res 18:821–829. doi:10.1101/gr.074492.107 18349386 PMC2336801

[4] Lin Y , Yuan J , Kolmogorov M , Shen MW , Chaisson M , Pevzner PA . 2016. Assembly of long error-prone reads using de Bruijn graphs. Proc Natl Acad Sci U S A 113:E8396–E8405. doi:10.1073/pnas.1604560113 27956617 PMC5206522

[5] Koren S , Walenz BP , Berlin K , Miller JR , Bergman NH , Phillippy AM . 2017. Canu: scalable and accurate long-read assembly via adaptive k-mer weighting and repeat separation . Genome Res 27:722–736. doi:10.1101/gr.215087.116 28298431 PMC5411767

[6] Wick RR , Judd LM , Gorrie CL , Holt KE . 2017. Unicycler: resolving bacterial genome assemblies from short and long sequencing reads. PLoS Comput Biol 13:e1005595. doi:10.1371/journal.pcbi.1005595 28594827 PMC5481147

[7] Wences AH , Schatz MC . 2015. Metassembler: merging and optimizing de novo genome assemblies. Genome Biol 16:207. doi:10.1186/s13059-015-0764-4 26403281 PMC4581417

[8] Tatusova T , DiCuccio M , Badretdin A , Chetvernin V , Nawrocki EP , Zaslavsky L , Lomsadze A , Pruitt KD , Borodovsky M , Ostell J . 2016. NCBI prokaryotic genome annotation pipeline. Nucleic Acids Res 44:6614–6624. doi:10.1093/nar/gkw569 27342282 PMC5001611

[9] Alcock BP , Raphenya AR , Lau TTY , Tsang KK , Bouchard M , Edalatmand A , Huynh W , Nguyen A-L , Cheng AA , Liu S , et al. . 2020. CARD 2020: antibiotic resistome surveillance with the comprehensive antibiotic resistance database. Nucleic Acids Res 48:D517–D525. doi:10.1093/nar/gkz935 31665441 PMC7145624

[10] Chen L , Yang J , Yu J , Yao Z , Sun L , Shen Y , Jin Q . 2005. VFDB: a reference database for bacterial virulence factors. Nucleic Acids Res 33:D325–D328. doi:10.1093/nar/gki008 15608208 PMC539962

[11] Li P , Zhang S , Wang J , Al-Shamiri MM , Han B , Chen Y , Han S , Han L . 2023. Uncovering the secretion systems of Acinetobacter baumannii: structures and functions in pathogenicity and antibiotic resistance. Antibiotics (Basel) 12:195. doi:10.3390/antibiotics12020195 36830106 PMC9952577

